# PepGMV *Rep*-Protein Expression in Mammalian Cells

**DOI:** 10.3390/v4091792

**Published:** 2012-09-24

**Authors:** Angela María Chapa-Oliver, Laura Mejía-Teniente, Teresa García-Gasca, Ramon Gerardo Guevara-Gonzalez, Irineo Torres-Pacheco

**Affiliations:** 1 CA Ingeniería de Biosistemas, División de Investigación y Posgrado, Facultad de Ingeniería, Universidad Autónoma de Querétaro, Cerro de las Campanas s/n, Santiago de Querétaro, Qro., 76010, México; Email: angellox02@gmail.com (A.M.C.-O.); laurayo@yahoo.com.mx (L.M.-T.); ramon.guevara@uaq.mx (R.G.G.-G.); 2 División de Investigación y Posgrado, Facultad de Ciencias Naturales, Universidad Autónoma de Querétaro, Avenida de las Ciencias s/n, Juriquilla, Qro., 76230, México; Email: tggasca@gmail.com

**Keywords:** *Geminivirus*, cell cycle, Rep protein

## Abstract

The *Geminiviruses* genome is a small, single strand DNA that replicates in the plant cell nucleus. Analogous to animal DNA viruses, *Geminiviruses* depend on the host replication machinery to amplify their genomes and only supply the factors required to initiate their replication. Consequently, *Geminiviruses* remove the cell-cycle arrest and induce the host replication machinery using an endocycle process. They encode proteins, such as the conserved replication-associated proteins (Rep) that interact with retinoblastoma-like proteins in plants and alter the cell division cycle in yeasts. Therefore, the aim of this work is to analyze the impact of *Pepper Golden Mosaic Virus* (PepGMV) Rep protein in mammalian cells. Results indicate that the pTracer-SV40:Rep construction obtained in this work can be used to analyze the Rep protein effect in mammalian cells in order to compare the cell cycle regulation mechanisms in plants and animals.

## 1. Introduction

*Geminiviruses* are plant pathogens which have small, single strand DNA genomes. They are characterized by their unique geminate capsids formed by two incomplete icosahedra [1,2]. *Geminiviruses* constitute a large family that is divided into the *Begomovirus, Mastrevirus*, *Curtovirus*, and *Topocuvirus* genera based on genome arrangement, insect vector, and host range [3,4]. The *Begomovirus* genus is the largest and includes whitefly-transmited, and bipartite (genome divided into two components) *geminivirus* [5]. *Pepper Golden Mosaic Virus *(*PepGMV*) belongs to *Begomovirus* genus and naturally infects pepper, tomato, tobacco, and other solanaceous crops. The typical symptoms induced by *PepGMV* in pepper plants, are yellow mosaic and wrinkled leaf and stunting of the plant [6]. 

*Geminiviruses* replicate in the cell nucleus via a rolling circle mechanism. They only supply the factors required to initiate their replication, and depend on the host replication machinery to amplify their genomes. Mammalian DNA-tumor viruses also employ this mechanism [1]. Many *Geminiviruses* replicate in differentiated cells that no longer contain detectable levels of DNA polymerases and associated factors [1,3]. Therefore, *geminiviruses* removed the cell-cycle arrest and thereby induce the host replication machinery in order to replicate their genomes [3,7,8].

Mammalian DNA-tumor viruses such as *polyoma*, *papilloma* and *adeno* viruses encode proteins that modify animal cell-cycle controls by binding to the retinoblastoma protein (pRB) or the related‑family members, p107 and p130 [9]. pRB-family members negatively regulate cell cycle progression and promote differentiation, in part, through their interactions with E2F transcription factors [10,11,12].

Analogous to animal DNA viruses, *Geminiviruses* encode proteins that interact with pRB proteins in plants. They encode for a conserved replication-associated protein (Rep) which is a multifunctional protein involved in viral replication, auto-regulation of its own gene transcription, and inactivation and recruitment of host-encoded proteins related to host DNA synthesis [4,13,14,15,16,17]. Rep is the only viral protein that is essential for viral replication. It initiates and terminates viral DNA synthesis and induces the accumulation of host-replication factors in infected cells [4,12]. Moreover, Rep protein interacts with host regulatory factors, including the proliferating cell nuclear antigen (PCNA), and the pRB, which modulates the plant cell cycle and differentiation [18,19]. Kittelmann *et al*. [2] found that *African Cassava Mosaic Virus* (ACMV) Rep protein expression in fission yeast (*Schizosaccharomyces pombe*) caused cell elongation that resembles *cdc* (cell division cycle) phenotypes. Cells expressing Rep protein increased DNA contents, suggesting that ACMV Rep protein promotes reinitiation of nuclear DNA replication during the fission yeast cell cycle [2]. 

Therefore, the objective of this work is to analyze the impact of *Pepper Golden Mosaic Virus* (PepGMV) Rep-protein expression in mammalian cell cycle regulation. To do this, the Rep open reading frame (ORF) was cloned into the expression vector pTracer-SV40 and 3T3L1 mouse fibroblast cells were transfected with the pTracer-SV40:Rep. Rep expression was analyzed by RT-PCR.

## 2. Results and Discussion

### 2.1. Expression Vector pTracer-SV40 and Ligation with PepGMV Rep-Protein Gene

**Figure 1:**
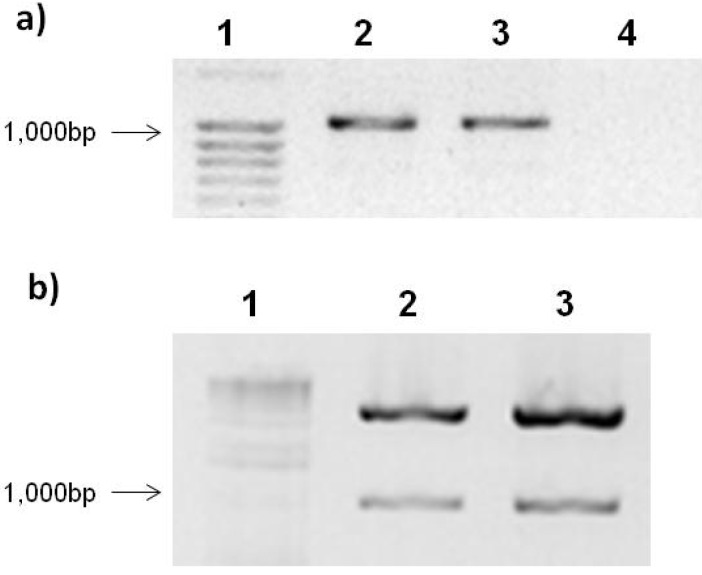
Agarose gel electrophoresis of Rep gene PCR amplification and construction double digestion.(**a**) PCR amplification of Rep gene. Lanes: 1, size molecular marker; 2, Rep; 3, RepATG^−^; 4, negative control. (**b**) Construction digestion with *SpeI* and *KpnI*. Lanes: 1, size molecular marker; 2, pTracerSV40:Rep; 3, pTracerSV40:RepATG^−^. The samples were loaded in a 1.2% agarose gel stained with EtBr.

**Figure 2:**
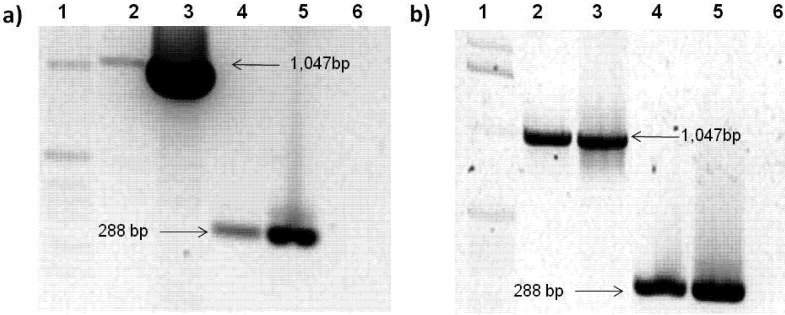
PCR amplification of *PepGMV* A component in transfected cells. (**a**) 48 h post-transfection. Lines: 1, size molecular marker; 2, Rep primers; 3, Rep primers positive control; 4, JM23 and JM24 primers; 5, JM23 and JM24 primers positive control; 6, negative control. (**b**) 6 days after transfection. Lines: 1, size molecular marker; 2, Rep primers; 3, Rep primers positive control; 4, JM23 and JM24 primers; 5, JM23 and JM24 primers positive control; 6, negative control. *PepGMV* A plasmid DNA was used as positive control. The samples were loaded in a 1.2% agarose gel stained with EtBr.

The *Rep* gene open reading frame (ORF) (GenBank: U57457.1) was amplified by PCR as shown in Figure 1a. Lines 2–4 (Figure 2a) show a band of approximately 1,047 bp corresponding to *PepGMV* Rep protein gene. After PCR amplification, Rep was cloned into the expression vector pTracer-SV40. Ligation was confirmed by double digestion with *SpeI* and *KpnI* enzymes. Figure 1b shows the gel electrophoresis of the double digestion product. As expected, two bands were observed in each of the samples. One corresponds to the linearized pTracerSV40 vector (Figure 1b, lines 2–4) which has a size of 4.2 kb and a second band corresponding to Rep (Figure 1b, lines 2–4).

These results confirmed the ligation of *PepGMV-*Rep gene into the pTracer-SV40 expression vector, obtaining the following constructions: pTracer-SV40:Rep and pTracer-SV40:RepATG^-^.

### 2.2. PepGMV Cell Transfection

**Figure 3:**
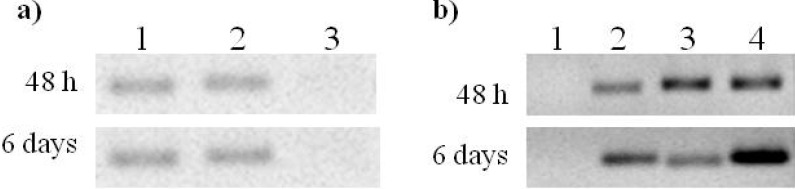
RT-PCR of *PepGMV* A transfected cells. (**a**) RT-PCR. Lines: 1, positive control; 2, GAPDH; 3, Rep. (**b**) RT-PCR re-amplification. Lines: 1, negative control; 2, GAPDH; 3, Rep; 4, positive control. The expected fragment size was 120 bp. Samples were loaded in a 2% agarose gel stained with EtBr.

In order to indicate if *Geminivirus PepGMV* is able to replicate in mammalian cells, the *PepGMV* A component was transfected into 3T3L1 fibroblast cells (as described in the Experimental section). *PepGMV* A DNA presence was confirmed by PCR amplification using the primers for the Rep gene and the JM23 and JM24 primers (Table 1). Figure 2 shows the PCR amplification of *PepGMV* A DNA-transfected cells. Again, a band of 1,047 bp corresponding to Rep is observed in samples taken at 24 and 144 h post transfection. Additionally, a band of 280 bp corresponding to the common region of *PepGMV* is observed, which confirms the presence of *Geminivirus* DNA inside the cells. In addition, RT-PCR was performed in order to verify Rep gene transcription in transfected cells (Figure 3a). The level of Rep transcription was too low to be clearly seen. Thus a re-amplification was made to corroborate the presence of the Rep transcript (Figure 3b). A band corresponding to Rep gene was observed after re-amplification, indicating that Rep transcription was taking place in transfected cells but with a low efficiency. Therefore geminivirus *PepGMV* was recognized by mammalian cell machinery and Rep transcription occurred.

**Table 1:** Oligonucleotides used for Rep gene PCR amplification, *PepGMV* A component detection and RT-PCR. Translation initiation codon is shown in bold letters. The underlined sequence corresponds to the Kozak sequence that was added in order to improve translation of Rep in mammalian cells.

Oligonuceotide name	Forward	Reverse	Fragment size
RepPK	GGTACCACCATGCCACTACCACCAAAATC	ACTAGTTTAGCTATCCTGTGCTGTGCT	1,047 bp
RepPA	GGTACCACCActGCACTACCACCAAAATC	ACTAGTTTAGCTATCCTGTGCTGTGCT	1,047 bp
JM23	TGGTGTAGGACTCCAGCAGAGTC		288 bp
JM24	TAGGCCCACACCTTGGTGACCAA		
β actin	AGGTATCCTGACCCTGAAGTACCCC	GGCCACACGCAGCTCATTGTA	106 bp
GAPDH	ATTGTTGCCATCAACGACCCC	CAAGCTTCCCATTCTCGGCC	116 bp
RepPepGMV	CAAAGCTGGTGATCCGAAAACG	GTTAAACGAGGATAATGGATAAGG	120 bp

**Figure 4:**
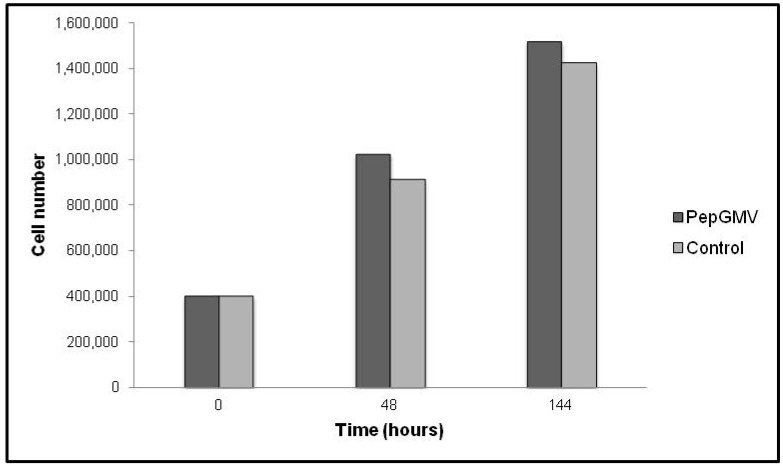
Transfected cells growth. Cells were counted using a hematocytometer after 48 and 144 h post transfection with *PepGMV* A component. The counting was performed in duplicate.

To determine if the Geminivirus affects the cell growth, transfected cells with *PepGMV* A component were counted. Figure 4 shows the number of cells at 0, 48 and 144 h after transfection. A difference in the cell number was observed in cells transfected with the *PepGMV* in contrast with control cells, indicating that the presence of *PepGMV* is affecting cell growth.

### 2.3. Rep-Gene Expression

**Figure 5:**
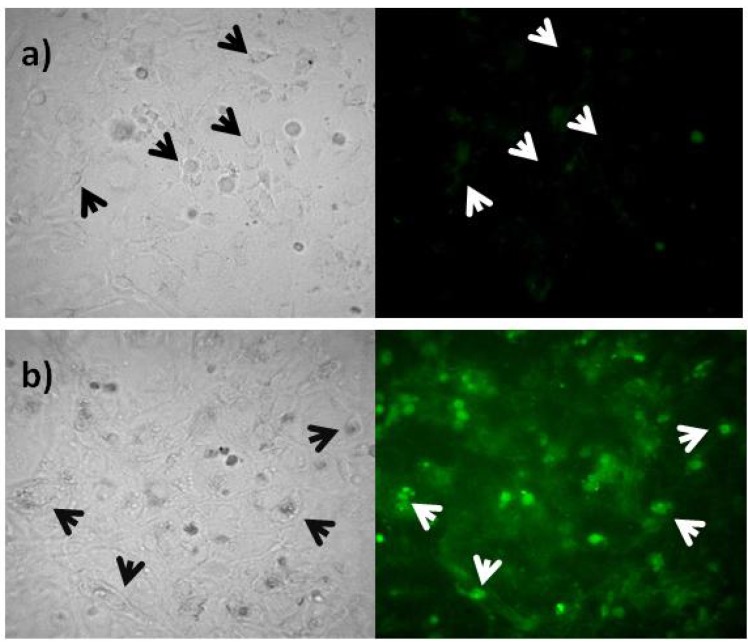
Transfected cells image under fluorescent microscopy. (**a**) Control cells. (**b**) Cells transfected with pTracer-SV40:Rep. Left: white light. Right: fluorescence. Arrows indicates cell position.

Expression of green fluorescent protein (GFP) was detected in order to verify the construction transfection into cells (Figure 5). Cells transfected with the pTracer-SV40:Rep (Figure 5a) reacted and fluoresced in contrast with control cells in which no fluorescence was observed (Figure 5b). This indicates the insertion of the construction and the expression of the GFP correlates with Rep expression.

**Figure 6:**
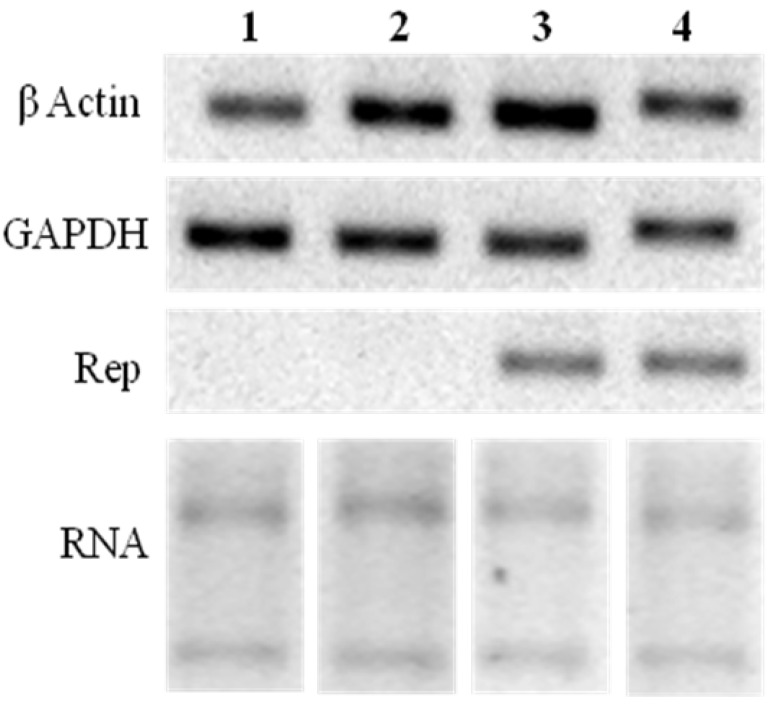
RT-PCR of transfected cells. Lines: 1, control cells; 2, cells transfected with the empty vector; 3, pTracer-SV40:Rep transfected cells; 4, pTracer-SV40:RepATG^−^ transfected cells. Samples were loaded in a 2% agarose gel stained with EtBr.

Additionally, Rep expression was confirmed by RT-PCR. Figure 6 shows the RT-PCR analysis of transfected cells at 72 h post transfection. A band corresponding to Rep gene was observed in cells transfected with both pTracer-SV40:Rep and pTracer-SV40:RepATG^−^ constructs. In contrast, with control and vector-transfected cells (Figure 6). This result indicates that Rep gene is being transcribed in transfected cells (Figure 6). 

## 3. Experimental Section

### 3.1. Viral Clone and Rep-Gene PCR Amplification

The dimeric clone of *Pepper Golden Mosaic Virus* (*PepGMV*) (Tamaulipas isolate) was used in this work. *PepGMV* genome has been previously described [6]. 

The Rep gene open reading frame (ORF) was amplified by PCR using the oligonucleotides shown in Table 1. The *PepGMV* Rep sequence used was obtained from the National Center for Biotechnology Information (NCBI) database (GenBank: U57457.1). To clone the Rep gene in the expression vector pTracer™-SV40, the sites for *SpeI* (extreme 3') and *KpnI* (extreme 5') restriction enzymes were used. PCR amplification conditions were as follows: 95 °C for 1 min, 30 cycles of 95 °C for 30 s and 68 °C for 2 min, and a final extension of 68 °C for 2 min. In addition, a negative translation control was generated by changing the Rep-gene initiation codon sequence (ATG) for the sequence ACT. 

### 3.2. Construction of pTracer™-SV40:Rep Expression Vector

After PCR amplification, the 1,047 bp gene was cleaned with the Wizard® PCR Preps DNA Purification System (Promega) and cloned in the intermediate pGem®-T Easy vector (Promega) using the manufacturer’s procedures. The pTracer™-SV40 vector (Invitrogen) was double digested with *SpeI* and *KpnI* enzymes (Fermentas) in order to linearize it and clone the Rep gene. Insert and vector purification was performed using the Silica Bead DNA Gel Extraction kit (Fermentas) and ligation reaction was done using the ligase T4 (Invitrogen) and a 4:1 insert/vector molar ratio. The insert presence was confirmed by double digestion with *SpeI* and *KpnI* enzymes (Fermentas).Gene correct orientation was confirmed by sequencing (Genetic Analyzer 3130, Applied Biosystems).

### 3.3. Cell Culture and Transfection

3T3L1 mice fibroblast cells (ATCC No. CL-173) were grown in Dubelco’s Modified Eagle Medium (DMEM, Gibco) supplemented with penicillin (62.1 mg/L; Sigma), streptomycin (100 mg/L; Sigma), anphotericin (250 µg/L; Gibco), and 5% of calf serum (Biowest). The cells grew in an incubator at 37 °C and a 5% CO_2_ atmosphere.

Cell transfection was performed by calcium-phosphate precipitation method [20]. Twenty four hours before transfection, cells were seeded into 60 mm plates at a concentration of 1 × 10^5^ cells/mL [20]. The culture medium was replaced with fresh medium one hour before transfection. The phosphate-DNA precipitation was performed as follows: 25 µg of plasmid DNA were mix with 100 µL of 2.5 M CaCl_2_. This solution was diluted with 1/10 TE buffer (1 mM Tris-HCl, 0.1 mM EDTA, pH 7.6) to a final volume of 1 mL. One volume of this 2× Ca/DNA solution was added drop by drop to an equal volume of 2× HEPES buffered saline solution. The resulting solution was incubated at room temperature for one minute prior to adding to the cells medium. One hundred microliters of precipitate per milliliter of culture medium were added to the cells Cells were exposed to the precipitate for 6 h at 37 °C and 5% CO_2_ atmosphere. After this time, the culture medium was replaced with fresh medium and the cells were incubated for 6 days at 37 °C and 5% CO_2_ atmosphere. Cells were transfected with pTracerSV40:Rep and pTracerSV40:RepATG^-^constructions. Additionally, the *PepGMV* A component was transfected in order to probe if *PepGMV* is able to replicate in mammalian cells. 

### 3.4. Rep Expression

3T3L1 cells transfection with pTracerSV40:Rep and pTracerSV40:RepATG^−^ was verified by detection of green fluorescent protein (GFP) by fluorescent microscopy. Additionally, Rep gene expression was verified by RT-PCR for treatments72 h after transfection. Total RNA was extracted from transfected cells using the SV total RNA isolation system (Promega).DNA first strand was generated by the First strand cDNA synthesis kit (Fermentas). The same treatment was applied to cells transfected with *PepGMV* A component. cDNA PCR amplification was performed with the oligonucleotides shown in Table 1 and the housekeeping genes glyceraldehyde-3-phosphate dehydrogenase (GADPH) and β actin were used as expression controls [21,22].

## 4. Conclusions

The *Pepper Golden Mosaic Virus* Rep gene was expressed in mammalian cells. This gene was cloned and transfected into mammalian cells. The expression level was at the same level as the housekeeping genes. Results indicated that the pTracer-SV40:Rep construction developed in this work can be used to analyze the Rep-protein expression in mammalian cells. Moreover, *PepGMV*, as *Geminivirus*, was recognized by mammalian-cell machinery and Rep transcription occurred. This trend indicates that this model can be used to study mammalian cell cycle transcriptome regulation using a *Geminivirus* member. The next step is to determine the effect of the Rep-expressed protein in the expression of mammalian cell cycle regulation genes.
